# Systematic comparison of RNA extraction techniques from frozen and fresh lung tissues: checkpoint towards gene expression studies

**DOI:** 10.1186/1746-1596-4-9

**Published:** 2009-03-24

**Authors:** Jai Prakash Muyal, Vandana Muyal, Brajesh Pratap Kaistha, Carola Seifart, Heinz Fehrenbach

**Affiliations:** 1 Clinical Research Group "Chronic Airway Diseases", Faculty of Medicine, Philipps-University Marburg, Marburg, Germany; 2 Dept. of Internal Medicine, Division of Nephrology, University Hospital Giessen & Marburg GmbH, Marburg, Germany; 3 Dept. of Internal Medicine, Division of Respiratory Medicine, University Hospital Giessen & Marburg GmbH, Marburg Hessen, Germany; 4 Section of Experimental Pneumology, Research Center Borstel, Leibniz Center for Medicine and Biosciences, Borstel, Germany; 5 School of Biotechnology, Gautam Buddha University, Greater Noida – 201308, India

## Abstract

**Background:**

The reliability of gene expression profiling-based technologies to detect transcriptional differences representative of the original samples is affected by the quality of the extracted RNA. It strictly depends upon the technique that has been employed. Hence, the present study aimed at systematically comparing silica-gel column (SGC) and guanidine isothiocyanate (GTC) techniques of RNA isolation to answer the question which technique is preferable when frozen, long-term stored or fresh lung tissues have to be evaluated for the downstream molecular analysis.

**Methods:**

Frozen lungs (n = 3) were prepared by long-term storage (2.5 yrs) in -80°C while fresh lungs (n = 3) were harvested and processed immediately. The purity and quantification of RNA was determined with a spectrophotometer whereas the total amounted copy numbers of target sequences were determined with iCycler detection system for assessment of RNA intactness (28S and 18S) and fragment sizes, i.e. short (GAPDH-3' UTR), medium (GAPDH), and long (PBGD) with 200 bp, 700 bp, and 1400 bp distance to the 3'ends of mRNA motif, respectively.

**Results:**

Total yield of RNA was higher with GTC than SGC technique in frozen as well as fresh tissues while the purity of RNA remained comparable. The quantitative reverse transcriptase-polymerase chain reaction data revealed that higher mean copy numbers of 28S and a longer fragment (1400 bp) were obtained from RNA isolated with SGC than GTC technique using fresh as well as frozen tissues. Additionally, a high mean copy number of 18S and medium fragment (700 bp) were obtained in RNA isolated with SGC technique from fresh tissues, only. For the shorter fragment, no significant differences between both techniques were noticed.

**Conclusion:**

Our data demonstrated that although the GTC technique has yielded a higher amount of RNA, the SGC technique was much more superior with respect to the reliable generation of an intact RNA and effectively amplified longer products in fresh as well as in frozen tissues.

## Background

DNA-microarray and quantitative reverse transcriptase-polymerase chain reaction (qRT-PCR) are two powerful techniques widely used in functional genomics for the analysis of gene expression profiles. These techniques are highly dependent on the quality of total RNA [1-3] obtained from either fresh or frozen biological samples.

RNA is a particularly labile bio-molecule and is much more susceptible to degradation by endogenous- and exogenous-nucleases and to non-specific degradation by divalent cations, heat, elevations in pH, and storage of tissue or cells over extended periods prior to RNA extractions, which result in falsely altered gene expression patterns [4]. Particularly, many efforts are currently being made to circumvent the problem associated with continues fragmentation and degradation of total RNA over-time. Total RNA can be isolated from archival tissue samples for example, by the formalin-fixed paraffin-embedded tissue-based technique [5]. However, this technique poses many problems due to the fact that formalin fixation cross-links nucleic acids and proteins. Further, mono-methylol is added to the amino groups for all four RNA bases (N-CH_2_OH) and subsequently methylene bridges are formed between neighbouring bases that resulted to continue degradation over-time [6]. Alternatively, immediate freezing of fresh tissue samples in liquid nitrogen and subsequent storage at -80°C until analysis preserves RNA integrity. However, this procedure is not routinely performed.

Obtaining high quality RNA is mandatory and depends upon the technique that has been employed [7]. Generally, the high quality of total RNA is assessed on the basis of RNA intactness and purity, respectively. The intactness of RNA is assessed by analyzing 28S and 18S subunits of ribosomal RNA either on micro-capillary chip's electrophoresis with fluorescent detection (Bioanalyzer, Agilent Technologies) or on ethidium stained agarose gel electrophoresis [8-11] while the purity is determined by calculating the ratio at absorption (A)_260 nm_/A_280 nm _by spectrophotometer [12]. Purity is considered to be adequate if the ratio is greater than 1.8 [13], and hence, recommended for introduction into the downstream molecular analysis.

Today, several other new techniques and kits are being offered for RNA isolation [14-17], which have their own respective principle and methodology and are believed to perform equally well. The two most reliable and widely used techniques for high throughput RNA isolation are 1) guanidine isothiocyanate-phenol:chloroform (GTC)-based RNA isolation technology and 2) Silica-gel column (SGC)-based RNA isolation technology. The GTC technique for isolation of RNA, which was developed by Chomczynski and Sacchi [18], is very popular because it requires much less time than other classical methods (e.g., CsCl_2 _ultracentrifugation). Moreover, GTC salt denatures the cellular proteins and inactivates RNases ensuring that isolated RNA is not degraded. Many commercial reagents (e.g. Trizol^®^, RNAzol™, RNAwiz™) are based on this principle. In contrast, the principle of SGC technology (Qiagen RNeasy Mini column) is a combination of the selective binding properties of a silica-based membrane with the speed of microspin technology, which allows saving time, money, and efficient use of small and precious biological samples. The comparison between GTC and SGC techniques are summarized in Table 1. Thus, when preparing the frozen or fresh samples for investigation of gene expression profiling, it is essential to briefly evaluate the technique of RNA isolation, especially in clinical applications with limited tissue material in terms of their reproducibility and reliability, respectively.

**Table 1:** Comparison of GTC-based versus SGC-based techniques

**GTC**	**SGC**
works best either with high (300 mg) or very low (20 mg) amount of tissue, because of phenol-chloroform follows by ethanol precipitation steps and uses of denaturants and RNase inhibitors	works best with low (<100 mg) amount of tissue, because filters may clog resulting in low yield of total RNA

Several chemical treatment steps in protocol may affect RNA quality	Limited steps in protocol may not have influences on RNA intactness

Complex protocol, takes 4 hrs	Simple protocol, takes only 20 min

chemically based aqueous-phase procedure, increased risk of cross contamination	No phase separation step present, low risk of cross contamination

Use of hazardous chemicals like phenol and chloroform requires specific safety rules in the laboratory	Absolutely safe while handling.

less expensive than SGC method	More expensive than GTC method

## Methods

### Experimental design

In the present study, we have tested a strategy to store lung tissues for the period of more than 2.5 yrs without any chemical treatment or processing, and investigated which RNA extraction technique (i.e. SGC and GTC) is preferable in terms of RNA recovery, purity, intactness, and amplification of various fragment sizes. A similar investigation was performed with fresh lung tissues.

Lung tissues were harvested from 4 months old male C57BL mice. Lung samples were either immediately dipped into liquid nitrogen, cut down into small fine pieces on sterile plate on ice using sterile blade and frozen at -80°C for 2.5 years until processing and analysis (n = 3 mice). Fresh lung tissue was processed and analysed immediately (n = 3 mice).

**Figure 1:**
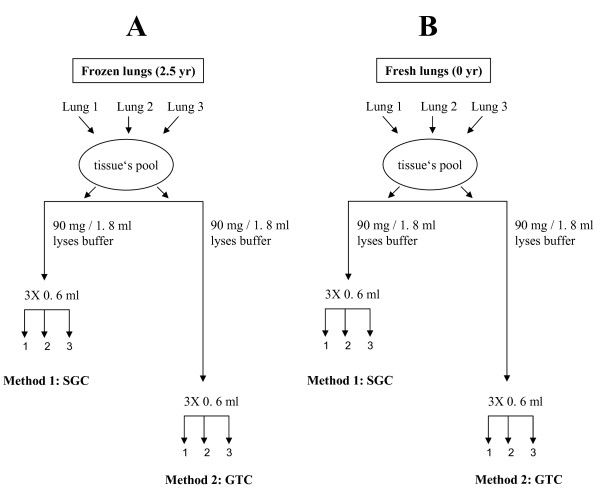
**Schematic representation of experimental plan**. A tissue pool has been created by combining lung 1, lung 2, and lung 3, respectively, which were obtained from either frozen or fresh samples. Pool of frozen or fresh tissue was divided in two equal parts, one for the SGC and another for the GTC technique. To test technique's reproducibility, each divided part was sub-divided into three identical parts (triplicate approach).

**Figure 2:**
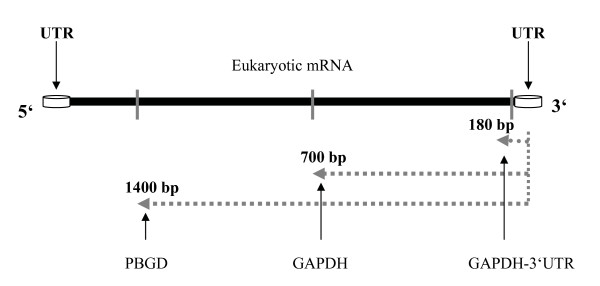
**Schematic representation of assessment for RNA intactness**. The location of the target sequences (i.e. GAPDH-3' UTR-200 bp, GAPDH-700 bp, and PBGD-1400 bp) on the transcripts.

The step by step experimental design for the systematic comparison of SGC and GTC techniques using frozen and fresh samples is given in Figure 1. Total RNA recovery was calculated from the RNA concentration measured by absorbance at 260 nm (A_260_) whereas purity was determined as per discussed above. The importance of RNA integrity and various fragment sizes were determined with qRT-PCR. For the assessment of RNA integrity, we used 28S and 18S subunits of ribosomal RNA, an internal control for RNA intactness. On the other hand, for the assessment of various fragment sizes of RNA, an amplification factor of Glyceraldehydes 3-phosphate dehydrogenase-3' un-translated region (GAPDH-3' UTR; short fragment), Glyceraldehyde 3-phosphate dehydrogenase (GAPDH; medium fragment) and Porphobilinogen deaminase (PBGD; long fragment), the respective distance of which to the 3'ends of messenger RNA (mRNA) were 200 bp, 700 bp, and 1400 bp (Figure 2), was taken in this study.

## Materials and methods

### Frozen- & Fresh- Lung Tissue Homogenization

The chopped pieces of lung tissues from frozen and fresh was collected in a 2.0 ml eppendorf tube, 1.9 ml of lyses buffer {RLT or 4 M GTC (4 M 25 mM Na-3-citrate: 0.5% laurylsarcosin, 100 mM Tris-Hcl)} was added, and with the help of a disposable syringe and a 20 G sterilized needle, tissue pieces were disrupted. The lysed-disrupted samples were kept at room temperature (RT) for 10 min, centrifuged for 15 min at 12,000 rpm to get rid off cellular debris, and supernatant was collected. In order to investigate the reproducibility of each method, collected supernatant was then divided into three equal volumes, i.e. 600 μl.

### Total cellular RNA Isolation, RNA Quantity, RNA purity, and RNA Quality Measurement

Collected supernatant was directly applied on the RNeasy column (SGC; Qiagen, Hilden, Germany) and total cellular RNA was isolated as per the steps given for the Qiagen RNA Miniprep kit. In contrast, using GTC technique collected supernatant was mixed with 200 μl of chloroform (Sigma-Aldrich, Steinheim, Germany) and 80 μl of 2 M NaoAc. The samples were vortexed and centrifuged at 4°C for 15 min at 12,000 rpm. The upper phase was transferred carefully into fresh 1.5 ml eppendorf tubes. For RNA precipitation, 500 μl of cold isopropanol (Sigma-Aldrich, Steinheim, Germany) with 70 μl of 2 M NaoAc was added to the supernatants and incubated for 2 hrs at -20°C. Samples were centrifuged at 4°C for 15 min at 12,000 rpm. The obtained pellets were washed with 500 μl of 70% chilled ethanol and centrifuged again at 4°C for 15 min at 12,000 rpm. The washed pellets were then air dried and finally dissolved in 40 μl of RNase free water.

The total RNA quantification and purity was determined with an Ultraspec 2100-spectrophotometer (Amersham Pharmacia Biotech, Cambridge, UK).

For total RNA quality determination, 1.2% of agarose gel was prepared by dissolving 1.2 g of agarose powder (Roth, Karlsruhe, Germany) in 100 ml of 1× Tris-Borate-EDTA buffer (Sigma-Aldrich, Steinheim, Germany). The mix was cooked until get boiled, and then 5 μl of 1% ethidium bromide (Roth, Karlsruhe, Germany) was added, mixed, and poured in to gel electrophoresis unit. RNA samples (1.0 μg each) were mixed with 2 μl of gel loading solution (Sigma-Aldrich, Steinheim, Germany) and were directly applied on a gel's wells. The integrity of total RNA was assessed on the basis of visualisation of 28S and 18S ribosomal RNA subunits under gel documentation system 2000 (Bio-Rad, München, Germany) as a band at positions 4.8 kb and 1.8 kb, respectively.

### First Strand cDNA Synthesis, Real-time PCR, and Analysis

First-strand cDNA synthesis was performed using 0.5 μg RNA in a total reaction of 20 μl using 1 μl oligo (dT)_12–18 _primer (Invitrogen, Karlsruhe, Germany) and Superscript™ II Reverse Transcriptase kit (Invitrogen, Karlsruhe, Germany). The reaction was incubated at 65°C for 5 min, at 37°C for 60 min, and at 72°C for 15 min in RoboCycler^® ^Gradient 40 (Startagene, Heidelberg, Germany). The cDNA qualities for all the samples were evaluated by performing GAPDH qualitative PCR (data not shown). The qRT-PCR for determining the amplification factor of target genes i.e. 28S, 18S, GAPDH-3' UTR, GAPDH, and PBGD were performed in a 96-well format iCycler Detection System (Bio-Rad, Hercules, USA). The reactions (20 μl) were set up with the SYBR™ Green PCR mix (ABgene^® ^Thermo Fischer Scientific, UK) according to the manufacturer's protocol. The thermal cycle conditions used for all reactions were as follows: Step 1: 95°C 15 min; followed by 40 cycles of Step 2: 95°C 50 sec; sequence-specific oligonucleotide primer's annealing temperature (Table 2) 40 sec; 72°C 40 sec; Step 3: 72°C 5 min. Due to the non-selective double stranded DNA binding of the SYBR™ Green I dye, melting curve analysis were performed to confirm the exclusive amplification of the expected PCR product.

**Table 2:** Primer details: Sequence, annealing temperature (AT), and amplicon size (AS) of target sequence

Target gene	Sequence (FP; 5' to 3')	AT [°C]	AS [bp]
			
	Sequence (RP; 5' to 3')		
GAPDH	AATGGTAAGGTCGGTGTGTGAA	60	262
			
	GAAGATGGTGATGGGCTTCC		

GAPDH-3' UTR	ACAGGGTGGTGGACCTCATG	60	102
			
	GTTGGGATAGGGCCTCTCTTG		

PBGD	TGCACGATCCTGAAACTCTG	60	163
			
	TGCATGCTATCTGAGCCATC		

28S	TCATCAGACCCCAGAAAAGG	60	102
			
	GATTCGGCAGGTGAGTTGTT		

18S	GCAATTATTCCCCATGAACG	60	123
	GGCCTCACTAAACCATCCAA		

The total amount of target gene copy numbers were determined by applying the formula 2 raised to the power mean of cycle threshold (2^-mean CT^). Unless stated otherwise, mean values ± s.d. are given. Since the aim of this study was to determine the most appropriate technique to isolate RNA, out of two tested techniques (i.e. SGC and GTC) in frozen- and fresh- lung samples, respectively, t-test (and non-parametric tests) was performed to determine the level of significance of differences between SGC and GTC. If normality and equal variance were not given at p > 0.1, Mann-Whitney rank sum test was used using GraphPad Prism 4 software program (San Diego CA, USA). Values of p < 0.05 were considered to be significant.

## Results

### Comparison of Two Techniques for Total RNA Isolation from Frozen and Fresh Lung Tissues

**Figure 3:**
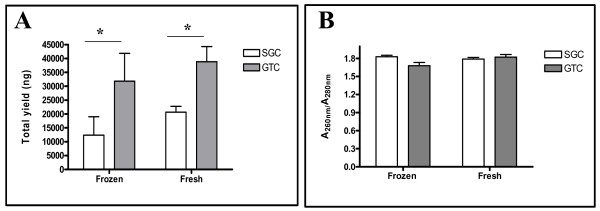
**Total yield and purity performance of RNA isolated from frozen and fresh lung tissues with SGC- and GTC-based techniques**. (A) Yield of total RNA in nanogram (ng), which was significantly higher in frozen and fresh lung tissue with GTC- than SGC-based isolation, respectively. (B) Total RNA purity assessment on the basis of A_260_/A_280_.

Firstly, the GTC and SGC techniques for RNA extraction were tested to assess, which technique is the most efficient and reproducible in terms of total yield and purity. RNA isolated with GTC technique was characterized by a significantly higher mean concentration {ng/total volume ( μl)} in comparison with RNA isolated by the SGC technique, which was true for both in frozen (p < 0.05; 2.6×) and in fresh (p < 0.05; 1.9×) lung tissues (Figure 3A).

The purity of the extracted RNA was comparable throughout the samples and was close to a ratio (A_260_/A_280_) of 1.8 with both, the GTC and the SGC technique, in frozen and fresh lung tissues, respectively (Figure 3B). A ratio close to 1.8 indicates that there were only limited protein contaminations.

### Quantitative PCR: Amplification factor

**Figure 4:**
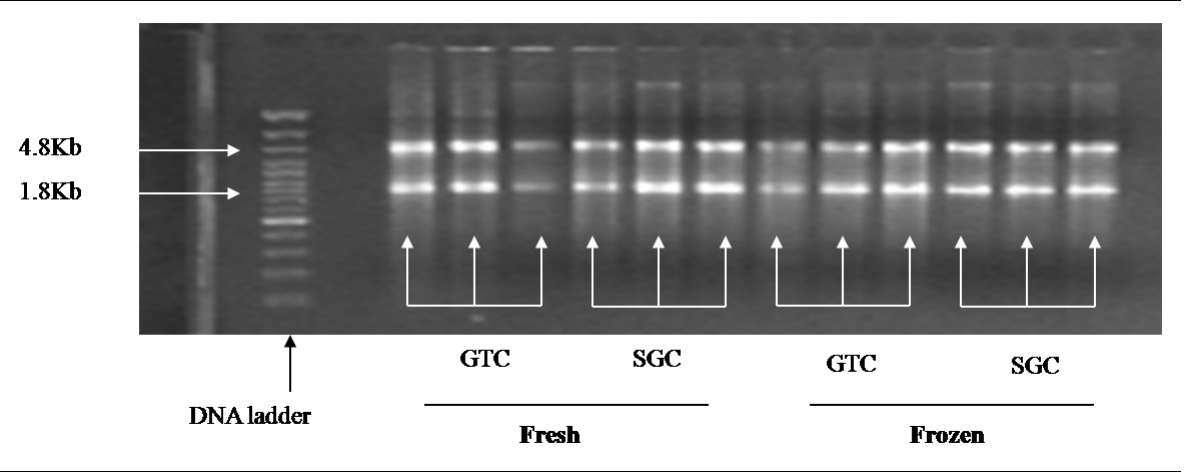
**Total RNA quality assessment on the basis of 18S and 28S rRNA**. On left side, lane # 1 shows DNA ladder (0.5 kb – 7.0 kb). The ethidium bromide-staining pattern of intact total RNA shows clearly defined 18S and 28S ribosomal RNA bands [lane # 3–5 (GTC; fresh); lane # 6–8 (SGC; fresh sample); lane # 9–11 (GTC; frozen); and lane # 12–14 (SGC; frozen).

Secondly, we evaluated the total RNA integrity on 1.2% agarose gel electrophoresis. All samples clearly demonstrated a visible intact band at two different positions, i.e. 4.8 kb and 1.8 kb, which represent 28S and 18S ribosomal RNA subunits, respectively (Figure 4).

**Figure 5:**
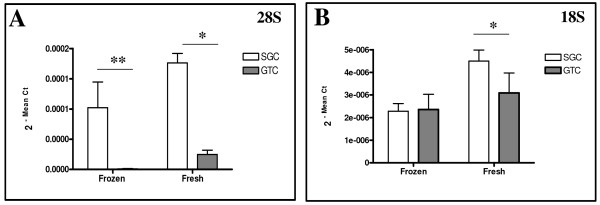
**Assessment of total RNA quality**. An amplification factor of 28S and 18S subunits of rRNA was determined; a higher copy numbers of 28S (A) was obtained with SGC method in frozen and fresh lung tissue compared with the GTC method. No significant difference was observed for 18S (B) in RNA isolated from frozen lung, however, more copy numbers of 18S was obtained from fresh lung tissues.

RNA integrity was further evaluated by determining an amplification factor of RNA's internal quality controls (i.e. 28S and 18S). qRT-PCR revealed that the mean copy numbers of 28S sequence was significantly higher in RNA isolated with the SGC compared to the GTC technique (Figure 5A). This difference was more pronounced in frozen (p < 0.01; 123×) than in fresh (p < 0.03; 7.1×) lung tissues. For the 18S sequence, RNA isolated from fresh lung tissues with SGC technique yielded higher mean copy numbers of 18S (p < 0.05; 1.6×) than RNA isolated from frozen lung tissues with GTC technique (Figure 5B).

**Figure 6:**
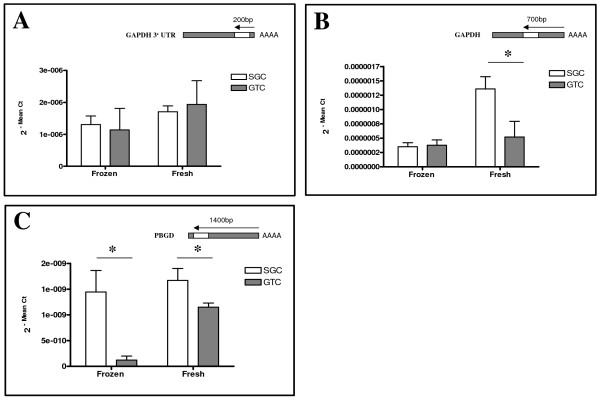
**Assessment of RNA fragment sizes**. An amplification factor of GAPDH-3'UTR, GAPDH, and PBGD was determined to assess the fragment sizes of RNA. The short (200 bp) and medium (700 bp) sequences were amplified by both techniques with similar fashion in RNA isolated from frozen and fresh lung tissue. More copy numbers of medium fragment (GAPDH) were obtained with fresh compared to frozen using SGC method. RNA isolated from SGC effectively amplified a longer fragment (<14,00 bp) in fresh and frozen lung tissue. In contrast, with the GTC technique a higher copy number of the longer product was obtained with fresh lung tissues only.

Lastly, to assess, which RNA extraction technique would reliably conserve a longer fragment from poly-A tail of mRNA, qRT-PCR was performed to determine the short (GAPDH-3' UTR, 200 bp), medium (GAPDH, 700 bp), and long (PBGD, 1400 bp) fragment sizes of RNA, respectively. The qRT-PCR for the GAPDH-3'UTR revealed that both techniques have the potential to amplify fragments of a size <200 bp in comparable manner (Figure 6A). RNA isolated with the SGC technique from fresh lung tissues exhibited significantly more copy numbers of the medium product (GAPDH) than RNA isolated with the GTC based technique (Figure 6B; p < 0.02; 2.6×), whereas no difference was seen in RNA extracted from frozen lung. The RNA fragment of 1400 bp of size (PBGD; longer) showed a clear reduction in the copy numbers of PBGD in RNA preparations with GTC than SGC using frozen (p < 0.05; 0.09×) and fresh (p < 0.02; 0.6×) lung tissues (Figure 6C). This indicates that smaller products were conserved when RNA samples were isolated with the GTC technique from frozen as well as fresh lung tissues compared to SGC based technique. Further, a higher and equivalent copy numbers of PBGD were obtained with the SGC-based method in frozen and fresh lung tissues than GTC technique.

## Discussion

The demand for high quality of RNA has been dramatically increased with the extensive application of DNA-microarray technology and real-time PCR-based expression profiling. A number of RNA extraction techniques are available to generate high quality RNA for use in gene expression profiling experiments [19]. Several attempts have been made to isolate RNA from formalin-fixed paraffin-embedded tissue using modifications of currently available techniques for RNA extraction [20]. However, the interpretation of results is often difficult and therefore, alternative methods for long-term storage of tissues prior to gene expression profiling are greatly required. The objective of the current study was to investigate which RNA extraction technique (i.e. GTC and SGC) is better and convenient if long-term stored frozen (2.5 yrs) and fresh lung tissue samples need to be evaluated for downstream applications. The comparison between the techniques was assessed in terms of RNA recovery, purity, intactness, and amplification of various fragment sizes of RNA (Figure 2).

The first step of evaluation after RNA extraction is the measurement of total recovery followed by the determination of purity and intactness of an isolated RNA. Our results demonstrate that irrespective of using RNA from frozen or fresh samples, the total recovery of RNA was higher with GTC- than SGC-based techniques (Figure 3A). Similar findings were obtained by Xiang and co-workers, who compared three different methods {i.e. (1) GTC, (2) Trifast, and (3) SGC techniques} for RNA extraction using sputum samples [21]. Our data are in accordance with their findings of the mean RNA concentration (i.e. recovery) obtained with the GTC-based technique being significantly higher than with the SGC-based technique with RNA purity being equivalent. We suggest that one reason for this difference in total yield may be the fact that the SGC technique uses a silica-gel membrane, which may get clogged with tissue lysate. Additionally, in the GTC-based technique phenol-chloroform and ethanol precipitation steps are implemented, which tend to concentrate RNA in a more robust manner than any other technique because a unique cocktail of denaturants and RNase inhibitors is used. This cocktail results into less degradation of RNA [22] and hence may contribute to increased total recovery compared to the SGC-based technique.

Next, the most important parameter in the evaluation procedure is to assess the intactness of RNA, which can be determined by analyzing the 18S and 28S subunits of ribosomal RNA on either the ethidium bromide-stained agarose gel [23,24] or micro-capillary electrophoresis traces [19,25]. Qualitative RNA analysis revealed clear bands of 28S and 18S in the agarose gel with no discernible difference in intensity or overall electrophoretic pattern between the different samples (Figure 4). We further validated RNA intactness by using qRT-PCR, because of its high sensitivity, good reproducibility, and the wide quantification range for determination of an amplification factor of 28S and 18S, respectively. Our qRT-PCR data revealed that SGC technique has a potential to generate higher copy numbers of 28S in RNA isolated from frozen and fresh lung tissues than GTC technique. On the other hand, a high copy number of 18S was observed in RNA isolated from fresh tissue with SGC technique only. We suggest that the capability to obtain high copy number of 28S (4.8 kb) and 18S (1.8 kb) with the silica gel-based technique may firstly relate to the selective adsorption to silica-gel membranes under controlled ionic conditions, which allows isolating RNA with high molecular size. Secondly, because the GTC technique does require the use of toxic chemicals like phenol and chloroform, and long protocol steps that may leads not to remove some contaminants likes phenol, chloroform, RNases, genomic DNA, and other chemicals, which are co-purified with RNA. Hence, these contaminants may interfere with the reverse transcription reaction process [26,27] and thereby resulted into loss of RNA population or indirectly copy numbers of tested sequences in contrast to the SGC technique.

In general, approximately 80 percent of the total RNA is ribosomal RNA and 15 percent is transfer RNA with protein-encoding messenger RNA constituting only a small portion (i.e. 5 percent) of the total RNA. To obtain an overall impression of RNA intactness, it is equally important to evaluate the quality of mRNA as well. By selecting three different sequences, which were classified according to their distance to the poly-A tail of mRNA, we asked to evaluate, which technique was able to conserve a longer fragment of mRNA from poly-A tail (3' to 5'). Our results demonstrated that any-one technique can be selected to isolate RNA either from frozen or fresh lung tissues for those sequences that are close to the poly-A tail of mRNA (<200 bp) and fragments lesser than 700 bp for downstream analysis (Figure 6A &6B).

Notably, the choice of the technique for RNA isolation had a significant impact on the potential to obtain appropriate copy numbers of bigger fragments as was demonstrated for the 1400 bp fragment of PBGD. In this respect, the SGC technique proved to be superior to the GTC technique both in RNA preparations from frozen and fresh lung tissues. The generation of high copy numbers of PBGD in RNA isolated from fresh as well as from frozen tissue suggests that the SGC-based technique is not only compatible with fresh but also with frozen tissues. We suggest that the reason for the superiority of the SGC technique again relates to the selective adsorption to silica-gel membranes and the avoidance of toxic chemicals and long-lasting protocol steps as discussed above.

## Conclusion

Comparing the SGC and GTC technique, we conclude that both techniques performed well in terms of total recovery and purity of RNA, isolated from 2.5 yrs old frozen and fresh lung tissues. With respect to RNA intactness, however, the SGC-based technique turned out to be superior to the GTC-based technique because of the conservation of an intact form of ribosomal RNA and conservation not only of short- to medium-sized but also of longer-sized RNA fragments. We may further conclude from our data that long-term storage of frozen lung tissues has only minor effects when compared to fresh lung tissues.

## Competing interests

The authors declare that they have no competing interests.

## Authors' contributions

JPM participated in the design of the study and performed qRT-PCR experiment, statistical analysis and drafted manuscript. VM carried out RNA isolation from frozen lung tissues, cDNA synthesis and qualitative PCR from frozen and fresh lung tissues. BPK carried out RNA isolation from fresh lung tissues. CS helped to analysis qRT-PCR data. HF conceived and supervised this work and helped to draft the manuscript.
